# Genetically determined high activities of the TNF-alpha, IL23/IL17, and NFkB pathways were associated with increased risk of ankylosing spondylitis

**DOI:** 10.1186/s12881-018-0680-z

**Published:** 2018-09-12

**Authors:** Jacob Sode, Steffen Bank, Ulla Vogel, Paal Skytt Andersen, Signe Bek Sørensen, Anders Bo Bojesen, Malene Rohr Andersen, Ivan Brandslund, Ram Benny Dessau, Hans Jürgen Hoffmann, Bente Glintborg, Merete Lund Hetland, Henning Locht, Niels Henrik Heegaard, Vibeke Andersen

**Affiliations:** 1 Institute of Regional Health Research, University of Southern Denmark, Odense, Denmark; 2 Department of Autoimmunology and Biomarkers, Statens Serum Institut, Copenhagen, Denmark; 3 Department of Rheumatology, Frederiksberg Hospital, Frederiksberg, Denmark; 4 Department of Rheumatology, Skåne University Hospital, Lund, Sweden; 5 Focused Research Unit for Molecular Diagnostic and Clinical Research, Hospital of Southern Jutland, Aabenraa, Denmark; 6 Medical Department, Viborg Regional Hospital, Viborg, Denmark; 7 National Research Centre for the Working Environment, Copenhagen, Denmark; 8 Microbiology and Infection Control, Statens Serum Institut, Copenhagen, Denmark; 9 Veterinary Disease Biology, University of Copenhagen, Copenhagen, Denmark; 10 Institute of Molecular Medicine, University of Southern Denmark, Odense, Denmark; 11 Department of Clinical Biochemistry, Herlev and Gentofte Hospital, Hellerup, Denmark; 12 Department of Biochemistry, Hospital of Lillebaelt, Vejle, Denmark; 13 Department of Clinical Microbiology, Slagelse Hospital, Slagelse, Denmark; 14 Department of Clinical Medicine, Aarhus University, Aarhus, Denmark; 15 Department of Respiratory Diseases B, Aarhus University Hospital, Aarhus, Denmark; 16 Department of Rheumatology, Gentofte and Herlev Hospital, Hellerup, Denmark; 17 The DANBIO Registry, Copenhagen Center for Arthritis Research, Center for Rheumatology and Spine Diseases, Rigshospitalet, Glostrup, Denmark; 18 Department of Clinical Medicine, Faculty of Health and Medical Sciences, University of Copenhagen, Copenhagen, Denmark; 19 Clinical Biochemistry, Clinical Institute, University of Southern Denmark, Odense, Denmark; 20 OPEN Odense Patient Data Explorative Network, Odense University Hospital, Odense, Denmark

**Keywords:** Ankylosing spondylitis, Single nucleotide polymorphism, SNP, Case-control study

## Abstract

**Background:**

Ankylosing spondylitis (AS) results from the combined effects of susceptibility genes and environmental factors. Polymorphisms in genes regulating inflammation may explain part of the heritability of AS.

**Methods:**

Using a candidate gene approach in this case-control study, 51 mainly functional single nucleotide polymorphisms (SNPs) in genes regulating inflammation were assessed in 709 patients with AS and 795 controls. Data on the patients with AS were obtained from the DANBIO registry where patients from all of Denmark are monitored in routine care during treatment with conventional and biologic disease modifying anti-rheumatic drugs (bDMARDs).

The results were analyzed using logistic regression (adjusted for age and sex).

**Results:**

Nine polymorphisms were associated with risk of AS (*p* < 0.05). The polymorphisms were in genes regulating a: the TNF-α pathway (*TNF* -308 G > A (rs1800629), and − 238 G > A (rs361525); *TNFRSF1A* -609 G > T (rs4149570), and *PTPN22* 1858 G > A (rs2476601)), b: the IL23/IL17 pathway (*IL23R* G > A (rs11209026), and *IL18–*137 G > C (rs187238)), or c: the NFkB pathway (*TLR1* 743 T > C (rs4833095), *TLR4* T > C (rs1554973), and *LY96–*1625 C > G (rs11465996)).

After Bonferroni correction the homozygous variant genotype of *TLR1* 743 T > C (rs4833095) (odds ratios (OR): 2.59, 95% confidence interval (CI): 1.48–4.51, *p* = 0.04), and *TNFRSF1A* -609 G > T (rs4149570) (OR: 1.79, 95% CI: 1.31–2.41, *p* = 0.01) were associated with increased risk of AS and the combined homozygous and heterozygous variant genotypes of *TNF* -308 G > A (rs1800629) (OR: 0.56, 95% CI: 0.44–0.72, *p* = 0.0002) were associated with reduced risk of AS.

**Conclusion:**

We replicated associations between AS and the polymorphisms in *TNF* (rs1800629), *TNFRSF1A* (rs4149570), and *IL23R* (rs11209026). Furthermore, we identified novel risk loci in *TNF* (rs361525), *IL18* (rs187238), *TLR1* (rs4833095), *TLR4* (rs1554973), and *LY96* (rs11465996) that need validation in independent cohorts. The results suggest that genetically determined high activity of the TNF-α, IL23/IL17, and NFkB pathways increase risk of AS.

## Background

Ankylosing spondylitis (AS) is a type of spondyloarthritis in which hallmark clinical features are inflammation at entheses and subchondral bone of the pelvic and spinal joints with subsequent abnormal new bone formation at these sites. Ultimately, this leads to ossification of entheses and joints resulting in loss of joint mobility. The incidence varies between 0.1 and 1.8% with the highest incidence in Scandinavia. Onset is typically in young adults with a male predominance. Medications used include non-steroid anti-inflammatory drugs (NSAIDs), and biological disease-modifying anti-rheumatic drugs (bDMARDs), i.e. tumor necrosis factor-α inhibitors (anti-TNF) and more recently also an interleukin(IL)-17A inhibitor (secukinumab) [1].

The cause of AS is unknown but is believed to involve a combination of genetic and environmental factors [2]. The heritability is polygenic and estimated to exceed 90%, with the HLA-B27 allele as the major contributor accounting for approximately 25% of the heritability of AS [2]. The IL-17/ IL-23 pathway and the TNF-α pathway are central in the pathogenesis of AS and alterations in these pathways have been shown in mouse models to affect development and severity of enthesitis [3, 4].

TNF-α can be activated by Pathogen-Associated Molecular Patterns (PAMPs) such as bacterial or viral DNA, flagellin, or lipopolysaccharide (LPS), through the NFkB pathway. PAMPs can be recognized by Toll-like receptors (TLRs) thereby initiating a kinase cascade which phosphorylates and degrades the NFkB inhibitor IkBα [5]. This releases NFkB which is transported from the cytosol to the nucleus where it initiates expression of pro- and anti-inflammatory cytokines including TNF-α and IL-17 (http://www.bu.edu/nf-kb/gene-resources/target-genes/). The TNF-α and NFkB pathway are intertwined and TNF-α can feedback stimulate NFkB by binding to TNF receptors (TNFR1 or TNFR2), resulting in a kinase cascade similar to, but distinct from, the pathway induced by TLRs [5].

The IL23/IL17 pathway can also stimulate TNF-α activity. The pro-inflammatory cytokine IL-17 enhances the production of other pro-inflammatory cytokines including TNF-α, and the secretion IL-17 itself can be enhanced by IL-23 [6].

PAMPs can also be recognized by intracellular Nod-like receptors (NLRs). In turn, NLRs can activate pro-inflammatory cytokines including IL-18 [7]. IL-18 is invloved in the IL23/IL17 pathway and can enhance the production of IL-17 [8].

The aim of this study was to assess whether functional single nucleotide polymorphisms.

(SNPs) in genes involved in the TNF-α, IL23/IL17, NFkB, and other pro- and anti-inflammatory pathways were associated with risk of AS.

## Methods

### Patients and samples

The DANBIO registry includes prospectively collected clinical data on patients with inflammatory joint diseases including smoking status, disease characteristics e.g. HLA-B27 status, disease activity, treatment, and treatment outcomes. Patients from all of Denmark are monitored in routine care during treatment with conventional and biologic disease modifying anti-rheumatic drugs (bDMARDs) [9].

Screening for tuberculosis before initiation of treatment with biological drugs is routinely performed in Denmark. Left over blood clots (after whole blood analysis for *Mycobacterium tuberculosis*) were collected from all patients screened for tuberculosis at Statens Serum Institut (Copenhagen, Denmark) from 01.09.2009 to 31.01.2013; the Department of Respiratory Diseases B and the Department of Clinical Microbiology, Aarhus University Hospital (Aarhus, Denmark) from 01.01.2011 to 31.01.2014; the Department of Clinical Biochemistry, Herlev and Gentofte Hospital (Hellerup, Denmark) from 01.03.2012 to 31.01.2014; the Department of Biochemistry, Hospital of Lillebaelt (Vejle, Denmark); and the Department of Biochemistry, Hospital of Slagelse (Slagelse, Denmark) from 01.01.2014 to 31.01.2014. Furthermore, from 01.01.2013 to 31.12.2013 blood samples were collected from all patients with AS treated with or without anti-TNF drugs at the Department of Rheumatology, Frederiksberg Hospital (Frederiksberg, Denmark).

By linking the unique personal identification number of Danish citizens (CPR-number) from each blood sample with the clinical data from DANBIO, 709 patients with AS (ICD-10: M45.9) were identified. The control group consisted of 795 healthy blood donors recruited from Viborg, Denmark.

### Genotyping

Fifty-one SNPs in genes involved in the TNF-α, IL23/IL17, NFκB, and other pro- and anti-inflammatory pathways were assessed. A list of all SNPs studied and genotype distribution is presented in Table 1 and SNPs associated with AS are summarized in Table 2.

**Table 1:** Odds ratios (OR) and 95% confidence interval (95CI) for genotypes studied among healthy controls and patients with ankylosing spondylitis (AS)

Gene rs-number	Healthy controls	AS	Unadjusted		Adjusted, age & sex		Adjusted, age, sex & smoking	
			OR (95 CI)	*p*	OR (9 5CI)	*p*	OR (95 CI)	*p*
*TLR1* rs4833095								
TT	485	415						
TC	261	238	1.07 (0.86–1.33)	0.57	1.03 (0.82–1.29)	0.83	1.05 (0.78–1.42)	0.73
CC	20	43	2.51 (1.45–4.34)	0.00095	2.59 (1.48–4.51)	0.00081	2.86 (1.44–5.68)	0.0026
TC or CC	281	281	1.17 (0.95–1.44)	0.15	1.14 (0.91–1.41)	0.25	1.18 (0.89–1.58)	0.26
*TLR2* rs3804099								
TT	241	197						
TC	393	354	1.10 (0.87–1.40)	0.42	1.07 (0.84–1.37)	0.58	1.02 (0.73–1.42)	0.90
CC	144	142	1.21 (0.89–1.63)	0.22	1.24 (0.91–1.68)	0.17	1.30 (0.87–1.96)	0.20
TC or CC	537	496	1.13 (0.90–1.41)	0.29	1.11 (0.89–1.40)	0.36	1.10 (0.80–1.50)	0.57
*TLR2* rs11938228								
CC	327	314						
CA	368	313	0.89 (0.71–1.10)	0.27	0.86 (0.69–1.07)	0.17	0.80 (0.60–1.08)	0.15
AA	76	69	0.95 (0.66–1.36)	0.76	0.92 (0.63–1.33)	0.66	1.03 (0.62–1.69)	0.92
CA or AA	444	382	0.90 (0.73–1.10)	0.30	0.87 (0.70–1.07)	0.19	0.84 (0.63–1.11)	0.22
*TLR2* rs4696480								
AA	199	179						
AT	417	348	0.93 (0.72–1.19)	0.55	0.89 (0.69–1.15)	0.38	0.84 (0.60–1.18)	0.31
TT	155	169	1.21 (0.90–1.63)	0.20	1.16 (0.86–1.58)	0.33	1.18 (0.78–1.78)	0.44
AT or TT	572	517	1.00 (0.79–1.27)	0.97	0.97 (0.76–1.23)	0.78	0.92 (0.67–1.27)	0.62
*TLR4* rs5030728								
GG	359	322						
GA	323	298	1.03 (0.83–1.28)	0.80	1.01 (0.81–1.27)	0.91	0.93 (0.69–1.25)	0.62
AA	78	70	1.00 (0.70–1.43)	1.00	0.98 (0.68–1.42)	0.93	0.87 (0.53–1.42)	0.57
GA or AA	401	368	1.02 (0.83–1.26)	0.83	1.01 (0.82–1.25)	0.94	0.91 (0.69–1.21)	0.53
*TLR4* rs1554973								
TT	440	395						
TC	272	261	1.07 (0.86–1.33)	0.55	1.06 (0.85–1.32)	0.62	0.98 (0.73–1.32)	0.90
CC	62	33	0.59 (0.38–0.92)	0.02	0.55 (0.34–0.86)	0.01	0.68 (0.38–1.23)	0.20
TC or CC	334	294	0.98 (0.80–1.21)	0.85	0.96 (0.78–1.19)	0.72	0.93 (0.70–1.24)	0.63
*TLR4* rs12377632								
TT	306	271						
TC	358	319	1.01 (0.81–1.26)	0.96	1.05 (0.84–1.32)	0.66	1.07 (0.78–1.46)	0.67
CC	102	96	1.06 (0.77–1.47)	0.71	1.11 (0.80–1.55)	0.52	1.41 (0.92–2.17)	0.12
TC or CC	460	415	1.02 (0.83–1.26)	0.86	1.06 (0.86–1.32)	0.58	1.14 (0.85–1.53)	0.37
*TLR5* rs5744168								
CC	672	605						
CT	94	89	1.05 (0.77–1.43)	0.75	1.05 (0.77–1.45)	0.74	0.89 (0.58–1.37)	0.60
TT	5	2	0.44 (0.09–2.30)	0.33	0.45 (0.08–2.43)	0.35	0.04 (0.00–3.54)	0.16
CT or TT	99	91	1.02 (0.75–1.39)	0.89	1.02 (0.75–1.40)	0.88	0.84 (0.55–1.29)	0.43
*TLR5* rs5744174								
TT	215	216						
TC	399	337	0.84 (0.66–1.07)	0.15	0.85 (0.67–1.09)	0.20	0.82 (0.60–1.14)	0.24
CC	144	138	0.95 (0.71–1.29)	0.76	1.02 (0.75–1.39)	0.91	0.87 (0.57–1.32)	0.51
TC or CC	543	475	0.87 (0.69–1.09)	0.23	0.90 (0.71–1.13)	0.36	0.84 (0.62–1.14)	0.26
*TLR9* rs187084								
TT	262	237						
TC	366	335	1.01 (0.80–1.27)	0.92	1.03 (0.82–1.31)	0.78	1.09 (0.79–1.50)	0.60
CC	142	120	0.93 (0.69–1.26)	0.66	0.91 (0.67–1.24)	0.56	1.07 (0.71–1.61)	0.76
TC or CC	508	455	0.99 (0.80–1.23)	0.93	1.00 (0.80–1.25)	0.98	1.08 (0.80–1.46)	0.60
*TLR9* rs352139								
GG	255	211						
GA	347	324	1.13 (0.89–1.43)	0.32	1.08 (0.85–1.38)	0.52	1.01 (0.73–1.40)	0.93
AA	167	139	1.01 (0.75–1.34)	0.97	0.96 (0.71–1.30)	0.79	0.80 (0.53–1.20)	0.27
GA or AA	514	463	1.09 (0.87–1.36)	0.45	1.04 (0.83–1.31)	0.72	0.94 (0.69–1.27)	0.68
*LY96* rs11465996								
CC	344	341						
CG	337	298	0.89 (0.72–1.11)	0.30	0.91 (0.73–1.14)	0.42	0.89 (0.66–1.20)	0.45
GG	81	53	0.66 (0.45–0.96)	0.03	0.68 (0.46–1.00)	0.0498	0.65 (0.39–1.10)	0.11
CG or GG	418	351	0.85 (0.69–1.04)	0.11	0.87 (0.70–1.07)	0.18	0.84 (0.63–1.12)	0.24
*CD14* Rs2569190								
GG	236	194						
GA	360	339	1.15 (0.90–1.46)	0.27	1.18 (0.92–1.51)	0.19	1.27 (0.91–1.78)	0.16
AA	170	157	1.12 (0.84–1.50)	0.43	1.20 (0.89–1.61)	0.24	1.46 (0.98–2.19)	0.06
GA or AA	530	496	1.14 (0.91–1.43)	0.26	1.18 (0.94–1.50)	0.15	1.32 (0.96–1.82)	0.08
*TIRAP* rs8177374								
CC	556	521						
CT	185	159	0.92 (0.72–1.17)	0.49	0.99 (0.77–1.27)	0.94	1.38 (0.99–1.91)	0.06
TT	21	15	0.76 (0.39–1.49)	0.43	0.76 (0.38–1.53)	0.45	1.31 (0.55–3.12)	0.55
CT or TT	206	174	0.90 (0.71–1.14)	0.39	0.97 (0.76–1.23)	0.81	1.38 (1.00–1.89)	0.047
*SUMO4* rs237025								
TT	215	195						
TC	362	358	1.09 (0.86–1.39)	0.48	1.08 (0.84–1.38)	0.55	1.04 (0.75–1.44)	0.80
CC	195	136	0.77 (0.57–1.03)	0.08	0.75 (0.55–1.01)	0.06	0.55 (0.36–0.84)	0.01
TC or CC	557	494	0.98 (0.78–1.23)	0.85	0.96 (0.76–1.22)	0.75	0.87 (0.64–1.19)	0.38
*NFKBIA* rs696								
GG	298	259						
GA	366	336	1.06 (0.85–1.32)	0.63	1.06 (0.84–1.33)	0.64	1.02 (0.75–1.39)	0.88
AA	101	90	1.03 (0.74–1.43)	0.88	0.97 (0.69–1.36)	0.86	1.07 (0.67–1.69)	0.78
GA or AA	467	426	1.05 (0.85–1.30)	0.65	1.04 (0.84–1.29)	0.73	1.03 (0.77–1.38)	0.84
*NFKB1* rs28362491								
Ins/Ins	269	258						
Ins/−	376	316	0.88 (0.70–1.10)	0.25	0.89 (0.70–1.12)	0.31	0.74 (0.54–1.01)	0.06
−/−	122	100	0.85 (0.62–1.17)	0.33	0.82 (0.59–1.13)	0.22	0.78 (0.51–1.19)	0.25
Ins/− or −/−	498	416	0.87 (0.70–1.08)	0.21	0.87 (0.70–1.08)	0.21	0.75 (0.56–1.01)	0.06
*TNF* rs1800629								
GG	527	549						
GA	223	129	0.56 (0.43–0.71)	0.0000032	0.58 (0.45–0.75)	0.000029	0.63 (0.45–0.89)	0.01
AA	25	9	0.35 (0.16–0.75)	0.01	0.39 (0.18–0.85)	0.02	0.19 (0.04–0.79)	0.02
GA or AA	248	138	0.53 (0.42–0.68)	0.00000030	0.56 (0.44–0.72)	0.0000047	0.59 (0.42–0.82)	0.0018
*TNF* rs361525								
GG	708	669						
GA	60	30	0.53 (0.34–0.83)	0.01	0.52 (0.32–0.82)	0.0049	0.61 (0.33–1.12)	0.11
AA	3	0	1.00 (1.00–1.00)	1.00	1.00 (1.00–1.00)	1.00	1.00 (1.00–1.00)	1.00
GA or AA	63	30	0.50 (0.32–0.79)	0.0027	0.49 (0.31–0.78)	0.0024	0.58 (0.32–1.05)	0.07
*TNFRSF1A* rs4149570								
GG	307	217						
GT	355	339	1.35 (1.07–1.70)	0.01	1.33 (1.05–1.68)	0.02	1.46 (1.06–2.00)	0.02
TT	109	132	1.71 (1.26–2.33)	0.00060	1.79 (1.31–2.46)	0.00027	2.26 (1.48–3.47)	0.00017
GT or TT	464	471	1.44 (1.16–1.78)	0.0010	1.44 (1.15–1.80)	0.0013	1.64 (1.21–2.22)	0.0014
*TNFAIP3* rs6927172								
CC	473	415						
CG	264	245	1.06 (0.85–1.32)	0.61	1.06 (0.85–1.33)	0.61	1.03 (0.76–1.39)	0.85
GG	40	25	0.71 (0.42–1.19)	0.20	0.70 (0.41–1.19)	0.18	0.51 (0.23–1.10)	0.09
CG or GG	304	270	1.01 (0.82–1.25)	0.91	1.01 (0.82–1.26)	0.91	0.95 (0.71–1.27)	0.73
*TGFB1* rs1800469								
CC	383	344						
CT	297	299	1.12 (0.90–1.39)	0.30	1.08 (0.87–1.35)	0.48	1.28 (0.95–1.71)	0.11
TT	86	53	0.69 (0.47–1.00)	0.047	0.69 (0.47–1.02)	0.06	0.69 (0.40–1.17)	0.17
CT or TT	383	352	1.02 (0.83–1.26)	0.83	1.00 (0.81–1.23)	0.97	1.14 (0.86–1.52)	0.35
*PTPN22* rs2476601								
GG	588	557						
GA	166	122	0.78 (0.60–1.01)	0.06	0.77 (0.59–1.00)	0.05	0.75 (0.52–1.09)	0.13
AA	11	6	0.58 (0.21–1.57)	0.28	0.57 (0.20–1.58)	0.28	0.83 (0.21–3.28)	0.80
GA or AA	177	128	0.76 (0.59–0.99)	0.04	0.76 (0.58–0.98)	0.04	0.76 (0.53–1.09)	0.13
*PPARG* rs1801282								
CC	548	511						
CG	207	167	0.87 (0.68–1.10)	0.23	0.85 (0.66–1.08)	0.18	0.87 (0.63–1.21)	0.42
GG	14	15	1.15 (0.55–2.40)	0.71	1.33 (0.62–2.83)	0.46	1.54 (0.60–3.98)	0.37
CG or GG	221	182	0.88 (0.70–1.11)	0.29	0.88 (0.69–1.11)	0.27	0.91 (0.67–1.26)	0.58
*IL1B* rs4848306								
GG	246	215						
GA	373	352	1.08 (0.85–1.36)	0.52	1.09 (0.86–1.39)	0.48	1.16 (0.84–1.60)	0.38
AA	151	125	0.95 (0.70–1.28)	0.72	0.96 (0.71–1.31)	0.81	0.88 (0.57–1.34)	0.55
GA or AA	524	477	1.04 (0.83–1.30)	0.72	1.06 (0.84–1.33)	0.64	1.08 (0.79–1.46)	0.64
*IL1B* rs1143623								
GG	401	365						
GC	316	278	0.97 (0.78–1.20)	0.76	0.98 (0.79–1.22)	0.87	1.07 (0.80–1.44)	0.66
CC	55	52	1.04 (0.69–1.56)	0.85	1.12 (0.74–1.69)	0.59	0.87 (0.48–1.57)	0.64
GC or CC	371	330	0.98 (0.80–1.20)	0.83	1.00 (0.81–1.24)	0.98	1.04 (0.78–1.38)	0.79
*IL1B* rs1143627								
TT	340	305						
TC	339	305	1.00 (0.81–1.25)	0.98	1.00 (0.79–1.25)	0.97	1.05 (0.78–1.42)	0.75
CC	97	86	0.99 (0.71–1.37)	0.94	1.01 (0.72–1.41)	0.95	0.85 (0.53–1.36)	0.50
TC or CC	436	391	1.00 (0.81–1.23)	1.00	1.00 (0.81–1.24)	1.00	1.00 (0.76–1.34)	0.97
*IL1RN* rs4251961								
TT	298	247						
TC	360	324	1.09 (0.87–1.36)	0.47	1.04 (0.83–1.32)	0.71	1.22 (0.89–1.67)	0.21
CC	112	105	1.13 (0.83–1.55)	0.44	1.05 (0.76–1.46)	0.76	1.41 (0.92–2.17)	0.12
TC or CC	472	429	1.10 (0.89–1.36)	0.40	1.05 (0.84–1.30)	0.68	1.26 (0.94–1.71)	0.12
*IL4R* rs1805010								
AA	209	201						
AG	410	317	0.80 (0.63–1.02)	0.08	0.79 (0.62–1.02)	0.07	0.73 (0.52–1.02)	0.07
GG	157	133	0.88 (0.65–1.19)	0.41	0.91 (0.67–1.24)	0.55	0.87 (0.58–1.33)	0.53
AG or GG	567	450	0.83 (0.66–1.04)	0.10	0.83 (0.65–1.05)	0.12	0.77 (0.56–1.06)	0.11
*IL6* rs10499563								
TT	476	439						
TC	259	225	0.94 (0.76–1.17)	0.60	0.94 (0.75–1.18)	0.60	0.77 (0.57–1.05)	0.10
CC	35	26	0.81 (0.48–1.36)	0.42	0.72 (0.42–1.25)	0.24	0.80 (0.39–1.63)	0.53
TC or CC	294	251	0.93 (0.75–1.14)	0.48	0.92 (0.74–1.14)	0.43	0.77 (0.57–1.04)	0.09
*IL6R* rs4537545								
CC	289	247						
CT	369	324	1.03 (0.82–1.29)	0.82	1.05 (0.83–1.32)	0.71	1.07 (0.79–1.47)	0.65
TT	117	113	1.13 (0.83–1.54)	0.44	1.18 (0.86–1.63)	0.30	1.17 (0.76–1.79)	0.48
CT or TT	486	437	1.05 (0.85–1.30)	0.64	1.08 (0.86–1.34)	0.51	1.09 (0.81–1.47)	0.55
*IL10* rs1800872								
CC	482	408						
CA	258	225	1.03 (0.83–1.29)	0.79	1.01 (0.80–1.27)	0.94	0.93 (0.68–1.26)	0.63
AA	35	42	1.42 (0.89–2.26)	0.14	1.35 (0.83–2.18)	0.22	1.47 (0.79–2.73)	0.22
CA or AA	293	267	1.08 (0.87–1.33)	0.50	1.05 (0.84–1.30)	0.67	0.99 (0.74–1.33)	0.95
*IL10* rs3024505								
CC	518	467						
CT	221	200	1.00 (0.80–1.26)	0.97	1.01 (0.80–1.28)	0.95	1.19 (0.87–1.61)	0.28
TT	22	24	1.21 (0.67–2.19)	0.53	1.32 (0.72–2.42)	0.37	1.80 (0.79–4.12)	0.16
CT or TT	243	224	1.02 (0.82–1.27)	0.84	1.04 (0.83–1.30)	0.76	1.23 (0.92–1.66)	0.17
*IL12B* rs3212217								
GG	499	460						
GC	235	200	0.92 (0.74–1.16)	0.49	0.95 (0.75–1.19)	0.64	0.94 (0.69–1.29)	0.72
CC	25	21	0.91 (0.50–1.65)	0.76	0.94 (0.51–1.72)	0.84	0.57 (0.23–1.41)	0.22
GC or CC	260	221	0.92 (0.74–1.15)	0.47	0.95 (0.76–1.19)	0.63	0.91 (0.67–1.23)	0.53
*IL12B* rs6887695								
GG	385	324						
GC	293	301	1.22 (0.98–1.52)	0.07	1.24 (0.99–1.55)	0.06	1.31 (0.97–1.77)	0.07
CC	72	70	1.16 (0.81–1.66)	0.43	1.16 (0.80–1.69)	0.43	0.98 (0.59–1.61)	0.94
GC or CC	365	371	1.21 (0.98–1.49)	0.07	1.22 (0.99–1.51)	0.06	1.24 (0.93–1.64)	0.14
*IL12RB1* rs401502								
CC	360	304						
CG	303	311	1.22 (0.98–1.51)	0.08	1.21 (0.96–1.51)	0.10	1.19 (0.88–1.61)	0.26
GG	87	70	0.95 (0.67–1.35)	0.79	0.97 (0.68–1.39)	0.87	1.18 (0.74–1.88)	0.48
CG or GG	390	381	1.16 (0.94–1.42)	0.17	1.15 (0.93–1.43)	0.19	1.19 (0.89–1.58)	0.24
*IL17A* rs2275913								
GG	340	307						
GA	336	301	0.99 (0.80–1.24)	0.94	0.98 (0.79–1.23)	0.89	0.90 (0.67–1.22)	0.51
AA	95	84	0.98 (0.70–1.36)	0.90	1.00 (0.71–1.40)	0.98	1.00 (0.63–1.57)	0.99
GA or AA	431	385	0.99 (0.80–1.22)	0.92	0.99 (0.80–1.22)	0.89	0.92 (0.69–1.22)	0.57
*IL18* rs187238								
GG	387	380						
GC	312	259	0.85 (0.68–1.05)	0.13	0.83 (0.66–1.03)	0.09	0.74 (0.55–1.00)	0.049
CC	64	41	0.65 (0.43–0.99)	0.04	0.69 (0.45–1.06)	0.09	0.58 (0.32–1.04)	0.07
GC or CC	376	300	0.81 (0.66–1.00)	0.0499	0.80 (0.65–0.99)	0.04	0.71 (0.53–0.95)	0.02
*IL18* rs1946518								
GG	282	259						
GT	363	329	0.99 (0.79–1.24)	0.91	0.96 (0.76–1.21)	0.71	0.89 (0.65–1.21)	0.45
TT	113	97	0.93 (0.68–1.29)	0.68	0.95 (0.68–1.31)	0.74	0.80 (0.51–1.24)	0.32
GT or TT	476	426	0.97 (0.79–1.21)	0.81	0.96 (0.77–1.19)	0.68	0.86 (0.64–1.16)	0.32
*IL23R* rs11209026								
GG	680	646						
GA	89	50	0.59 (0.41–0.85)	0.0045	0.63 (0.43–0.91)	0.02	0.64 (0.38–1.05)	0.08
AA	5	1	1.00 (1.00–1.00)	1.00	1.00 (1.00–1.00)	1.00	1.00 (1.00–1.00)	1.00
GA or AA	94	51	0.57 (0.40–0.82)	0.0021	0.60 (0.42–0.87)	0.01	0.63 (0.38–1.03)	0.06
*IFNG* rs2430561								
TT	199	181						
TA	398	369	1.02 (0.80–1.30)	0.88	1.01 (0.79–1.30)	0.92	1.08 (0.77–1.52)	0.65
AA	161	139	0.95 (0.70–1.29)	0.74	0.97 (0.71–1.32)	0.85	1.09 (0.72–1.64)	0.68
TA or AA	559	508	1.00 (0.79–1.26)	0.99	1.00 (0.79–1.27)	0.99	1.08 (0.79–1.50)	0.62
*IFNGR1* rs2234711								
TT	290	232						
TC	361	348	1.20 (0.96–1.51)	0.11	1.20 (0.95–1.51)	0.12	1.15 (0.84–1.57)	0.40
CC	119	108	1.13 (0.83–1.55)	0.43	1.09 (0.79–1.50)	0.60	1.11 (0.72–1.70)	0.65
TC or CC	480	456	1.19 (0.96–1.47)	0.12	1.17 (0.94–1.46)	0.16	1.14 (0.84–1.53)	0.40
*IFNGR2* rs8126756								
TT	553	522						
TC	168	130	0.82 (0.63–1.06)	0.13	0.83 (0.64–1.09)	0.18	0.86 (0.60–1.24)	0.42
CC	18	12	0.71 (0.34–1.48)	0.36	0.69 (0.32–1.49)	0.35	0.53 (0.18–1.54)	0.24
TC or CC	186	142	0.81 (0.63–1.04)	0.09	0.82 (0.64–1.06)	0.13	0.83 (0.59–1.17)	0.28
*IFNGR2* rs17882748								
CC	199	173						
CT	391	341	1.00 (0.78–1.29)	0.98	1.00 (0.77–1.30)	0.99	1.01 (0.71–1.42)	0.97
TT	153	174	1.31 (0.97–1.76)	0.08	1.31 (0.97–1.78)	0.08	1.16 (0.77–1.73)	0.48
CT or TT	544	515	1.09 (0.86–1.38)	0.48	1.09 (0.86–1.39)	0.48	1.05 (0.76–1.45)	0.76
*TBX21* rs17250932								
TT	526	497						
TC	210	179	0.90 (0.71–1.14)	0.39	0.94 (0.74–1.19)	0.61	0.84 (0.60–1.17)	0.30
CC	32	19	0.63 (0.35–1.12)	0.12	0.66 (0.36–1.19)	0.17	0.37 (0.14–0.98)	0.046
TC or CC	242	198	0.87 (0.69–1.08)	0.21	0.90 (0.72–1.14)	0.39	0.78 (0.56–1.07)	0.12
*NLRP1* rs2670660								
AA	222	202						
AG	390	328	0.92 (0.73–1.18)	0.52	0.96 (0.75–1.23)	0.73	1.12 (0.80–1.56)	0.52
GG	154	154	1.10 (0.82–1.47)	0.53	1.11 (0.82–1.49)	0.51	1.12 (0.75–1.67)	0.59
AG or GG	544	482	0.97 (0.78–1.22)	0.82	1.00 (0.79–1.26)	0.98	1.11 (0.81–1.52)	0.50
*NLRP1* rs878329								
GG	217	206						
GC	394	333	0.89 (0.70–1.13)	0.34	0.89 (0.69–1.14)	0.35	0.99 (0.71–1.38)	0.93
CC	155	155	1.05 (0.79–1.41)	0.73	1.05 (0.78–1.41)	0.75	1.03 (0.69–1.54)	0.90
GC or CC	549	488	0.94 (0.75–1.17)	0.57	0.93 (0.74–1.18)	0.56	1.00 (0.73–1.36)	0.98
*NLRP3* rs10754558								
CC	294	248						
CG	355	324	1.08 (0.86–1.36)	0.50	1.06 (0.84–1.34)	0.61	1.10 (0.81–1.51)	0.54
GG	111	116	1.24 (0.91–1.69)	0.18	1.25 (0.91–1.71)	0.17	1.11 (0.71–1.72)	0.65
CG or GG	466	440	1.12 (0.90–1.39)	0.30	1.11 (0.89–1.38)	0.36	1.11 (0.82–1.49)	0.51
*NLRP3* rs4612666								
CC	435	360						
CT	280	277	1.20 (0.96–1.49)	0.11	1.23 (0.99–1.54)	0.07	1.28 (0.95–1.72)	0.10
TT	53	48	1.09 (0.72–1.66)	0.67	1.19 (0.78–1.82)	0.41	1.07 (0.59–1.94)	0.82
CT or TT	333	325	1.18 (0.96–1.45)	0.12	1.23 (0.99–1.52)	0.06	1.24 (0.94–1.65)	0.13
*CARD8* rs2043211								
AA	321	298						
AT	342	316	1.00 (0.80–1.24)	0.97	0.98 (0.79–1.23)	0.89	0.90 (0.67–1.22)	0.50
TT	94	78	0.89 (0.64–1.25)	0.52	0.89 (0.63–1.26)	0.50	0.91 (0.57–1.44)	0.68
AT or TT	436	394	0.97 (0.79–1.20)	0.80	0.96 (0.78–1.19)	0.72	0.90 (0.67–1.19)	0.45
*JAK2* rs12343867								
TT	398	358						
TC	299	263	0.98 (0.79–1.22)	0.84	0.96 (0.76–1.20)	0.69	0.82 (0.61–1.12)	0.21
CC	61	65	1.18 (0.81–1.73)	0.38	1.11 (0.75–1.63)	0.61	1.03 (0.62–1.71)	0.91
TC or CC	360	328	1.01 (0.82–1.25)	0.90	0.98 (0.79–1.21)	0.86	0.86 (0.64–1.14)	0.29

**Table 2:** Biological interpretation of the single nucleotide polymorphisms (SNPs) associated with ankylosing spondylitis (AS)

Gene	Rs-number	Pathway	Model	OR (95% CI)	*P*-value / Bonferroni^a^	Effect of minor-allele	Biological interpretation
*TLR1*	rs4833095	Pathogen recognition	CC vs TT	2.59 (1.48–4.51)	0.00081 / 0.04	743C increase TLR1 level in PBMC [56]	Increased TLR1 level was associated with increased risk of AS. This could indicate that a genetically determined high activity of the NFkB pathway, and thus high TNF-α and IL-17 activity, was associated with increased risk of AS.
*TLR4*	rs1554973	Pathogen recognition	CC vs TT	0.55 (0.34–0.86)	0.010 / 0.51	Unknown [67]	–
*LY96*	rs11465996	Pathogen recognition	GG vs CC	0.68 (0.46–1.00)	0.049 / 1.00	-1625G increase MD-2 and TNF-α levels in human U937 cells and whole blood leukocytes [57]	Increased MD-2 and TNF-α level was associated with a reduced risk of AS. In contrast to the other results this indicate that genetically determined high TNF-driven inflammatory response was associated with reduced risk of AS.
*TNF*	rs1800629	Cytokines	GA or AA vs GG	0.56 (0.44–0.72)	0.0000047 / 0.00024	-308A increase expression in jurkat cells [65], reduce mRNA level in PBMC and serum [48] or no association was found [49]	Reduced TNF-α mRNA level was associated with reduced risk of AS. This could indicate that genetically determined high TNF-driven inflammatory response was associated with increased risk of AS.
*TNF*	rs361525	Cytokines	GA or AA vs GG	0.49 (0.31–0.78)	0.0024 / 0.12	-238A reduce expression in PBMC [49]	Reduced TNF-α expression was associated with reduced risk of AS. This indicates that genetically determined high TNF-driven inflammatory response was associated with increased risk of AS.
*TNFRSF1A*	rs4149570	Cytokines	GT or TT vs GG	1.44 (1.15–1.80)	0.0013 / 0.066^b^	-609 T increase expression in PBMC [50]	Increased TNF-α receptor 1 expression was associated with increased risk of AS. This indicates that genetically determined high TNF-driven inflammatory response was associated with increased risk of AS.
*PTPN22*	rs2476601	Immune response	GA or AA vs GG	0.76 (0.58–0.98)	0.037 / 1.00	1858A reduce TNF-α level in serum [51]	Reduced TNF-α level was associated with reduced risk of AS. This indicates that genetically determined high TNF-driven inflammatory response was associated with increased risk of AS.
*IL18*	rs187238	Cytokines	GC or CC vs GG	0.80 (0.65–0.99)	0.044 / 1.00	-137C reduce IL-18 level in serum [53] and expression in PBMC [54]	Reduced IL-18 expression, and thus reduced IL-17 and TNF-α activity, was associated with reduced risk of AS. This indicates that a genetically determined high activity of the IL23/IL17 pathway was associated with increased risk of AS.
*IL23R*	rs11209026	Cytokines	GA or AA vs GG	0.60 (0.42–0.87)	0.0071 / 0.36	rs11209026A reduce IL-17 level in PBMC [52]	Reduced IL-17 level was associated with reduced risk of AS. This indicates that a genetically determined high activity of the IL23/IL17 pathway was associated with increased risk of AS.

*OR* Odds ratio

*95% CI* 95% confidence interval

*PBMC* peripheral blood mononuclear cell

^a^The Bonferroni calculations were based on the 51 SNPs assessed in this study

^b^The *TNFRSF1A* (rs4149570) TT vs GG: OR: 1.79, 95% CI: 1.31–2.41, *p* = 0.00027, Bonferroni = 0.014

DNA extraction (Maxwell 16 LEV Blood DNA Kit; Promega, Madison, WI, USA) was performed as described by *Bank* et al. [10]. For the healthy controls, DNA was extracted from EDTA-stabilized peripheral blood by either PureGene (Qiagen, Hilden, Germany) or Wizard Genomic (Promega, Madison, Wisconsin, USA) DNA purification kit according to the manufacturers` instructions [11–17]. Competitive Allele-Specific Polymerase chain reaction (KASP™), an end-point PCR technology, was used by LGC Genomics for genotyping (LGC Genomics, Hoddesdon, United Kingdom) (http://www.lgcgenomics.com/).

### Power calculation

The Genetic Power Calculator was utilized for power analysis of discrete traits (http://zzz.bwh.harvard.edu/gpc/cc2.html). The lowest minor allele frequency (MAF) of the studied SNPs was 0.10. The ‘high-risk allele frequency’ was set to 0.10, the ‘prevalence’ was set to 0.0018 [18], D-prime was set to 1, type I error rate was set to 0.05 and number of cases and control:case ratio was 795:709. This cohort study had more than 80% chance of detecting a dominant effect with an odds ratio (OR) of 1.4 for AS.

### Statistical analysis

Logistic regression was used to compare genotype distributions among patients with AS versus healthy controls. Crude odds ratio, odds ratio adjusted for age and sex, and odds ratio adjusted for age, sex, and smoking status were assessed (Table 1). A chi-square test was used to test for deviation from Hardy-Weinberg equilibrium in the healthy controls and for haplotype analysis (Tables 3, 4, 5 and 6).

**Table 3:** Association of the *TLR2* haplotype combinations and risk of ankylosing spondylitis (AS). The haplotype combinations in *TLR2* described 93% of the genotypes observed

Haplotype combinations	Haplotypes			N_AS_ (%)	N_Control_ (%)	OR^a^	(95% CI)	*P*-value
	rs4696480 A > T	rs11938228 C > A	rs3804099 T>C^b^					
11	T:T	A:A	T:T	69 (11)	76 (10)	1.00	–	–
22	A:A	C:C	C:C	72 (11)	74 (10)	1.07	0.68–1.70	0.82
33	A:A	C:C	T:T	28 (4)	34 (5)	0.91	0.50–1.65	0.76
44	T:T	C:C	C:C	14 (2)	10 (1)	1.52	0.64–3.70	0.38
12	T:A	C:A	C:T	158 (24)	197 (27)	0.88	0.60–1.30	0.55
13	T:A	C:A	T:T	76 (12)	103 (14)	0.81	0.52–1.26	0.37
14	T:T	C:A	C:T	59 (9)	49 (7)	1.33	0.80–2.19	0.31
23	A:A	C:C	C:T	77 (12)	89 (12)	0.95	0.61–1.49	0.91
24	T:A	C:C	C:C	52 (8)	55 (8)	1.04	0.63–1.72	0.90
34	T:A	C:C	C:T	51 (8)	44 (6)	1.28	0.76–2.14	0.43

*OR* Odds ratio

^a^OR was calculated for each haplotype combination by using the haplotype 11 as reference group

^b^The variant allele of rs3804099T T > C has been shown to decrease TNF-α, IL-1β & IL-6 level [68]

**Table 4:** Association between *TLR4* haplotype combinations and risk of ankylosing spondylitis (AS). The haplotype combinations in *TLR4* described 94% of the genotypes observed

Haplotype combinations	Haplotypes			N_AS_ (%)	N_Control_ (%)	OR^a^	(95% CI)	*P*-value
	rs12377632 T > C	rs1554973 T > C	rs5030728 G > A					
11	C:C	T:T	G:G	95 (14)	101 (14)	1.00	–	–
22	T:T	T:T	A:A	69 (10)	74 (10)	0.99	0.64–1.53	1.00
33	T:T	C:C	G:G	29 (4)	57 (8)	0.54	0.32–0.92	0.03
44	T:T	T:T	G:G	3 (0)	5 (1)	0.64	0.15–2.74	0.72
12	T:C	T:T	G:A	154 (23)	188 (25)	0.87	0.61–1.24	0.47
13	T:C	T:C	G:G	126 (19)	129 (17)	1.04	0.72–1.51	0.85
14	T:C	T:T	G:G	30 (5)	32 (4)	1.00	0.56–1.77	1.00
23	T:T	T:C	G:A	99 (15)	106 (14)	0.99	0.67–1.47	1.00
24	T:T	T:T	G:A	31 (5)	24 (3)	1.37	0.75–2.51	0.36
34	T:T	T:C	G:G	28 (4)	26 (4)	1.14	0.63–2.09	0.76

*OR* Odds ratio

The biological effect of the three polymorphisms in *TLR4* was unknown

^a^OR was calculated for each haplotype combination by using the haplotype 11 as reference group

**Table 5:** Association between *IL1B* haplotype combinations and risk of ankylosing spondylitis (AS). The haplotype combinations in *IL1B* described 97% of the genotypes observed

Haplotype combinations	Haplotypes			N_AS_ (%)	N_Control_ (%)	OR^a^	(95% CI)	*P*-value
	rs4848306 -3737G > A [69, 70]	rs1143623 -1464G > C [69, 71]	rs1143627 -31 T > C [69, 71, 72]					
11	A:A	G:G	T:T	125 (18)	148 (20)	1.00	–	–
22	G:G	C:C	C:C	52 (8)	54 (7)	1.14	0.73–1.79	0.65
33	G:G	G:G	T:T	32 (5)	41 (5)	0.92	0.55–1.55	0.79
44	G:G	G:G	C:C	5 (1)	3 (0)	1.97	0.46–8.42	0.48
12	A:G	G:C	T:C	163 (24)	185 (24)	1.04	0.76–1.43	0.81
13	A:G	G:G	T:T	141 (20)	147 (19)	1.14	0.82–1.58	0.50
14	A:G	G:G	T:C	44 (6)	38 (5)	1.37	0.84–2.25	0.26
23	G:G	C:G	C:T	84 (12)	92 (12)	1.08	0.74–1.58	0.70
24	G:G	C:G	C:C	28 (4)	34 (4)	0.98	0.56–1.70	1.00
34	G:G	G:G	T:C	14 (2)	16 (2)	1.04	0.49–2.21	1.00

*OR* Odds ratio

The variant allele of −3737 G > A [69], −1464 G > C [70] and − 31 T > C [71, 72] have been shown to decrease IL-1β level [69–72]

^a^OR was calculated for each haplotype combination by using the haplotype 11 as reference group

**Table 6:** Association of the *TNF* haplotype combinations and risk of ankylosing spondylitis (AS). The haplotype combinations in *TNF* described 97% of the genotypes observed

Haplotype combinations	Haplotypes		N_AS_ (%)	N_Control_ (%)	OR^a^	(95% CI)	*P*-value
	rs361525 G>A^b^	rs1800629 G>A^c^					
11	G:G	G:G	523 (76)	469 (61)	1.00	–	–
22	G:G	A:A	9 (1)	25 (3)	0.32	(0.15–0.70)	0.005
12	G:G	G:A	125 (18)	210 (28)	0.53	(0.41–0.69)	< 0.0001
13	G:A	G:G	26 (4)	47 (6)	0.50	(0.30–0.81)	0.007
14	G:A	G:A	4 (1)	12 (2)	0.30	(0.10–0.93)	0.05

*OR* Odds ratio

^a^OR was calculated for each haplotype combination by using the haplotype 11 as reference group

^b^The variant allele of TNF -238A rs361525A G > A has been shown to reduce expression of TNF-α [49]

^c^The variant allele of TNF -308A rs1800629 G > A has been shown to reduce mRNA level [48]

Statistical analyses were performed using STATA version 15 (StataCorp LP, College Station, TX, USA).

## Results

### Study population

Among the patients with AS the median age was 32 years (SD: 11.5) and 68% (483/709) were males. The healthy controls had a median age of 43 years (SD: 11.5) and 52% (411/384) were males. Among the patients 37% (118/323), 23% (73/323), and 41% (132/323) and among the controls 26% (207/788), 24% (189/788), and 50% (392/788) were current smokers, former smokers and never smokers, respectively. HLA-B27 staus was available for 498 patients of which 83% (411/498) were positive. Sixty percent (427/709) of the patients were treated with anti-TNF.

The genotype distributions among the healthy controls deviated from Hardy-Weinberg equilibrium for *TLR1* (743 T > C (rs4833095)) (*p* = 0.03), *TLR2* (− 16,934 A > T (rs4696480)) (*p* = 0.02), *TLR4* (rs1554973 T > C) (*p* = 0.03), *TLR9* (1174 G > A (rs352139)) (*p* = 0.02) and *TGFB1* (− 509 C > T (rs1800469)) (*p* = 0.02). After correction for multiple testing, all SNPs studied were in Hardy-Weinberg equilibrium.

### Polymorphisms associated with susceptibility of AS

In the age and sex adjusted analysis, the homozygous variant genotype of *TLR1* 743 T > C (rs4833095) (OR: 2.59, 95% CI: 1.48–4.51, *p* = 0.0008) and the combined homozygous and the heterozygous variant genotypes of *TNFRSF1A* -609 G > T (rs4149570) (OR: 1.44, 95% CI: 1.15–1.80, *p* = 0.001) were associated with increased risk of AS. The homozygous variant genotype of *TLR4* T > C (rs1554973) (OR: 0.55, 95% CI: 0.34–0.86, *p* = 0.01) and *LY96–*1625 C > G (rs11465996) (OR: 0.68, 95% CI: 0.46–1.00, *p* = 0.05), and the combined homozygous and the heterozygous variant genotypes of *TNF* -308 G > A (rs1800629) (OR: 0.56, 95% CI: 0.44–0.72, *p* = 0.000005), *TNF* -238 G > A (rs361525) (OR: 0.49, 95% CI: 0.31–0.78, *p* = 0.002), *PTPN22* 1858 G > A (rs2476601) (OR: 0.76, 95% CI: 0.58–0.98, *p* = 0.04), *IL18*–137 G > C (rs187238) (OR: 0.80, 95% CI: 0.65–0.99, *p* = 0.04), and *IL23R* G > A (rs11209026) (OR: 0.60, 95% CI: 0.42–0.87, *p* = 0.01) were associated with reduced risk of AS (Table 1).

After Bonferroni correction for multiple testing the homozygous variant genotype of *TLR1* 743 T > C (rs4833095) (OR: 2.59, 95% CI: 1.48–4.51, *p* = 0.04) and *TNFRSF1A* -609 G > T (rs4149570) (OR: 1.79, 95% CI: 1.31–2.41, p = 0.01) were associated with increased risk of AS and the combined homozygous and the heterozygous variant genotypes of *TNF* -308 G > A (rs1800629) (OR: 0.56, 95% CI: 0.44–0.72, *p* = 0.0002) were associated with reduced risk of AS (Table 2).

SNPs associated with AS and the biological effect of the SNPs are summarized in Table 2.

### Haplotype analysis

Haplotype analyses of *TLR2*, *TLR4, IL1B* and *TNF* are shown in Tables 3, 4, 5 and 6, respectively.

The *TLR4* haplotype combination 33 (rs12377632TT, rs1554973CC and rs5030728GG) was associated with reduced risk of AS (OR: 0.54, 95% CI: 0.32–0.92, *p* = 0.03) compared to the haplotype combination 11. In *TNF* all haplotype combinations were associated with reduced risk of AS compared to the haplotype combination 11 (rs361525GG and rs1800629GG).

No associations were found for haplotype combinations of *TLR2* or *IL1B*.

## Discussion

In this case-control study, polymorphisms in a: the TNF-α (*TNF* (rs1800629 and rs361525), *TNFRSF1A* (rs4149570), and *PTPN22* (rs2476601)), b: the IL23/IL17 (*IL23R* (rs11209026), and *IL18* (rs187238)), or c: the NFkB (*TLR1* (rs4833095), *TLR4* (rs1554973), and *LY96* (rs11465996)) pathways were associated with risk of AS.

The found assocaitions for *TNF* (rs1800629) [19–22], *TNFRSF1A* (rs4149570) [23], and *IL23R* (rs11209026) [24–33] are in agreement with other case-control studies. Furthermore, Zhao et al. found that the variant allele of *NLRP3* (rs4612666) was associated with increased risk of AS in Chinese patients [23]. In our study we found a trend for associations of the variant allele of *NLRP3* (rs4612666) with increased risk of AS (*p* = 0.06). However, our results are in contrast to a meta-analysis of the *PTPN22* (rs2476601) polymorphism that did not find an association with AS [34]. Finally, we identified novel risk loci in *TNF* (rs361525), *IL18* (rs187238), *TLR1* (rs4833095), *TLR4* (rs1554973), and *LY96* (rs11465996) that need validation in independent cohorts.

Most of the SNPs assessed in our study have known biological effects thus allowing a biological interpretation of the observed associations based on increased or reduced gene activity as summarized in Table 2 [35–47]. The associations observed for the *TNF* (rs1800629 and rs361525) polymorphisms suggest that reduced TNF-α mRNA level and expression of TNF-α was associated with reduced risk of AS [48, 49]. This is supported by our haplotype analysis which also suggests that the variant alleles of *TNF* rs1800629 and rs361525 were associated with reduced risk of AS. Likewise, the associations observed for the *TNFRSF1A* (rs4149570) polymorphism indicates that increased expression of the TNF-α receptor 1 was associated with increased risk of AS [50]. Furthermore, the associations observed for the *PTPN22* (rs2476601) polymorphism suggests that reduced TNF-α serum level was associated with reduced risk of AS [51]. Taken together, this suggests that genetically determined high activity of the TNF-α pathway was associated with increased risk of AS.

IL-17 is known to induce the production of many cytokines including TNF-α [6]. IL-18 is a pro-inflammatory cytokine known to enhance the production of IL-17, TNF-α, and IL-1β [8]. In this study, the association observed for the *IL23R* (rs11209026) polymorphism suggests that reduced IL-17 serum level, and thus reduced TNF-α activity, was associated with reduced risk of AS [52]. Furthermore, the associations observed for the *IL18* (rs187238) polymorphism indicates that reduced IL-18 expression, and thus reduced IL-17 and TNF-α activity, was associated with reduced risk of AS [53, 54]. The associations found in the *IL23R* (rs11209026) and the *IL18* (rs187238) polymorphisms thus suggest that a genetically determined high activity of the IL23/IL17 pathway was associated with increased risk of AS. The two SNPs furthermore support that genetically determined high activity of the TNF-α pathway was associated with increased risk of AS. The observed associations between the polymorphisms in *IL23R* and *IL18* and risk of AS are in line with previous studies pointing out the IL23/IL17 pathway as central to the pathophysiology of AS [3, 4, 55].

This study also suggests that the NFkB pathway may be involved in the etiology of AS. The associations observed for the *TLR1* (rs4833095) polymorphism suggests that increased TLR1 level was associated with increased risk of AS [56]. High level of TLR1 may lead to increased NFkB activation and thus increased TNF-α and IL-17 activity, which is in line with the other results. However, in contrast to the other results*,* the associations observed for the *LY96* (rs11465996) polymorphism suggests that increased MD-2 (*LY96)* and TNF-α level was associated with a reduced risk of AS [57]. Finally, the *TLR4* (rs1554973) polymorphism was associated with reduced risk of AS which was supported by the haplotype results (Table 4). The biological effect of the *TLR4* (rs1554973) polymorphism is unknown, however, the result supports the notion that the NFkB pathway may be involved in the etiology of AS.

Both TNF-α [58] and interleukin-17 inhibitors [59] have been shown to reduce inflammation and improve symptoms in patients with AS [60]. Furthermore, increased levels of TNF-α, IL-17, IL-23, IL-1β, and IL-6 have been found in sera and synovial fluid from AS patients [61–64]. The genetic associations between AS and the polymorphisms in *TLR1*, *TLR4*, *LY96*, *TNF*, *TNFRSF1A*, *IL18*, and *IL23R* found in this study, could potentially – in part – explain this altered cytokine milieu present in AS patients.

There are aspects of this study which should be interpreted with care. Conflicting results have been reported for the *TNF* (rs1800629) polymorphism [48, 49, 65]. Furthermore, the *TNF* polymorphisms, as well as the HLA-B27 locus, are located on chromosome 6, and there is a risk that even a minor linkage disequilibrium could have confounded our results [2]. *TLR1* (rs4833095), *TLR2* (rs4696480), *TLR4* (rs1554973), *TLR9* (rs352139), and *TGFB1* (rs1800469) were not in Hardy-Weinberg equilibrium among the healthy controls. Due to the number of polymorphisms analyzed this is probably a type II error. The polymorphisms do not deviate from Hardy-Weinberg equilibrium when corrected for multiple testing. We cannot exclude that some of our positive findings may be due to chance due to the obtained *p*-values and the number of statistical tests performed. When the results were corrected for multiple testing only the variant allele of *TLR1* (rs4833095) and *TNFRSF1A* (rs4149570) were associated with increased risk of AS and the variant allele of *TNF* (rs1800629) was associated with reduced risk of AS.

A major strength of this study was that the cohort was rather large including 709 patients with AS and 795 healthy controls and the associations that we report were biologically plausible. Also, the validity of the diagnosis is expected to be high, since the patients were identified via a clinical database that the rheumatologist use for prospective monitoring of patients as part of routine care [66].

## Conclusions

In conclusion, we replicated associations between AS and the polymorphism *TNF* (rs1800629), *TNFRSF1A* (rs4149570), and *IL23R* (rs11209026). Furthermore, we identified novel risk loci in *TNF* (rs361525), *IL18* (rs187238), *TLR1* (rs4833095), *TLR4* (rs1554973), and *LY96* (rs11465996) that need validation in independent cohorts. The results suggest that genetically determined high activity of the TNF-α, IL23/IL17, and NFkB pathways increase the risk of AS.
